# Left Atrial Appendage Exclusion in Atrial Fibrillation Radiofrequency Ablation during Mitral Valve Surgery: A Single-Center Experience

**DOI:** 10.1155/2021/9999412

**Published:** 2021-08-02

**Authors:** C. Lavalle, M. Straito, E. Chourda, S. Poggi, G. Frati, W. Saade, A. G. M. Marullo, M. V. Mariani, M. Magnocavallo, F. Miraldi

**Affiliations:** ^1^Cardio Thoracic-Vascular and Organ Transplantation Surgery Department, Policlinico Umberto I Hospital, Rome, Italy; ^2^Department of Clinical Internal, Anaesthesiology and Cardiovascular Sciences, “Sapienza” University of Rome, Rome, Italy; ^3^Department of Medico-Surgical Sciences and Biotechnologies, “Sapienza” University of Rome, Rome, Italy; ^4^IRCCS NEUROMED, Pozzilli, Italy

## Abstract

**Background:**

Atrial fibrillation surgical radiofrequency ablation (AFSA) during mitral valve surgery (MVS) has almost completely superseded the Cox-Maze procedure for the treatment of atrial fibrillation.

**Methods:**

We retrospectively analyzed 100 patients who underwent MVS + AFSA in our institution from January 2008 to June 2017. We compared the effectiveness of AFSA in patients who underwent LAA exclusion to those who did not. Moreover, we analyzed the role of preoperative AF duration (≤ or >1 year) and medial-lateral left atrial dimensions (ML-LAD) (≤ or >6 cm). The efficacy endpoint was freedom from AF at discharge and at 2-year follow-up. The safety endpoints were need of a permanent pacemaker (PMK), surgical re-exploration, occurrence of stroke, and left circumflex artery or esophageal lesions.

**Results:**

Overall, the rate of AF freedom was 69% at discharge and 80% at 2-year follow-up. LAA exclusion did not influence AF freedom at 2-year follow-up, and 84.6% of patients who underwent LAA exclusion were in the sinus rythm (SR) at 2 year compared to 75% of those who did not receive LAA exclusion free from AF as well (*p*=0.230). AF duration ≤1 or >1 year did not influence sinus rhythm (SR) maintenance (85.7% vs. 75.8%; *p*=0.224), and in these two groups, LAA exclusion did not change the efficacy of AFSA. ML-LAD ≤ 6 cm was associated with better results in terms of SR maintenance. A statistically significant association between LAA exclusion and SR maintenance at 2-year follow-up (*p*=0.017) was found among patients with ML-LAD ≤ 6 cm. Complications included 7 cases of PMK implantation, 2 cases of surgical re-exploration, and 1 case of stroke. No circumflex artery or esophageal lesions occurred after surgical procedures.

**Conclusions:**

In our experience, AFSA during isolated MVS resulted in good outcomes in terms of SR maintenance and incidence of complications. AF duration ≤ 1 year did not influence results, while patients with ML-LAD ≤ 6 cm had significantly better results regarding SR at follow-up. In patients with ML-LAD ≤ 6 cm, LAA exclusion significantly increased the success rate of SR maintenance at 2-year follow-up.

## 1. Introduction

Mitral valve (MV) disorders are associated with atrial enlargement, which predisposes to atrial fibrillation (AF) [1]. AF is maintained by multiple micro-reentrant circuits that require a critical mass. On this basis, in 1987, Dr. James Cox completed the first effective AF surgical ablation (AFSA), using a technique named Maze I [2, 3]. A “cut and sew” technique created a labyrinth (or maze) of electrically silent injuries which, in addition to the isolation of the arrhythmogenic foci (localized predominantly in the pulmonary veins, PVs) [4, 5], resulted in successful interruption of the AF micro-reentrant circuits. With the same rationale, the use of radiofrequency (RF) [6, 7], instead of a scalpel, made AFSA simpler and less dangerous. AFSA is mainly indicated for patients who are eligible for mitral valve surgery (MVS) [8] with the aim of restoring and/or maintaining sinus rhythm (SR), reducing symptoms and the risk of stroke, [9] and improving survival and cardiac functions (class I, level A) [8–11]. As a result, the rate of AFSA combined with MVS has increased from 53% to 61.5% over the last few years [1, 12].

There is still no agreement on the lesion set to perform; therefore, each operator uses their own lesion scheme based on personal experience [13]. The crista terminalis, ligament of Marshall, coronary sinus, and left atrial appendage (LAA) may play a role in AF physiopathology and may represent ablation targets. Increasing evidence has suggested that LAA electrical exclusion or isolation might play a role in reducing AF burden, especially in persistent or long-standing persistent AF [14] defined according to the international guidelines [15] as episodes that last more than 7 days or where the patient requires pharmacological, electrical, or ablation procedures (persistent) and episodes that last for more than 1 year (long-standing persistent).

The aim of this study is to evaluate the added value of LAA exclusion in terms of long-term SR maintenance in patients undergoing AFSA during MVS.

Safety endpoints were the occurrence of stroke, need of a permanent pacemaker (PMK), left circumflex artery or esophageal lesions, and surgical re-exploration.

## 2. Materials and Methods

**Figure 1:**
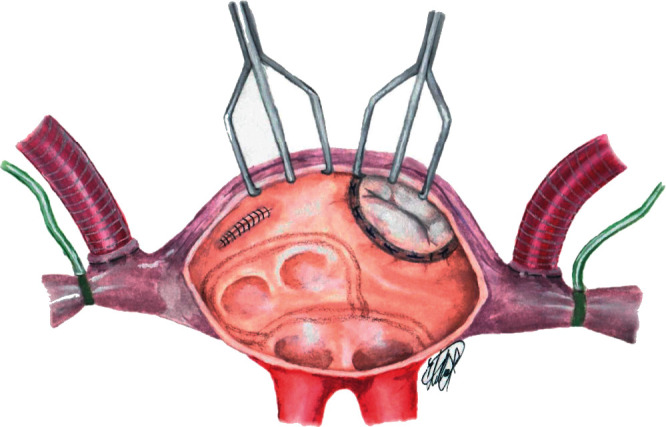
Left atrial lesion set and surgical LAA exclusion.

We retrospectively analyzed 100 patients with isolated mitral valve disease who underwent MVS combined with endocardial AFSA from January 2008 to June 2017. Patients with indication for cardiac surgery procedures other than MVS and the history of previous catheter ablation for atrial fibrillation were excluded. Duration of AF and systolic dysfunction were not considered as exclusion criteria. All patients underwent preoperative transthoracic and intraoperative transesophageal echocardiographic evaluations before surgery to examine the mitral valve anatomy, to plan the surgical strategy, and, at procedure completion, to evaluate any residual mitral insufficiency. All patients presented severe mitral valve disease (severe mitral regurgitation: 81% and mitral stenosis: 19%) (Table 1). Patients with mitral stenosis underwent MV replacement. When mitral valve repair was possible, it was accomplished according to the “French correction” and/or implantation of neochordae whenever necessary. All patients underwent MV repair or replacement and combined RF ablation following the “left-only” ablation scheme (Figure 1) through a standard median sternotomy access.

Surgical procedures were carried out on moderate hypothermia with aortic and bicaval cannulation for extracorporeal circulation and cardioplegic arrest with Buckberg cold blood antegrade cardioplegia and warm reperfusion. In all cases, the left atrium was accessed through an incision behind the Waterston groove in front of the right PVs. Two right atrial and one right ventricular temporary pacing wires were placed after aortic cross-clamp removal, in case intra and/or postoperative stimulation was deemed necessary.

MVS was always performed before AFSA. In our scheme, the radiofrequency ablation lines (Figure 1) were performed twice in all patients, with encircling of the PVs and exclusion of the posterior left atrial wall starting from the atriotomy lesion associated or not to LAA exclusion according to surgeon preference. When performed, LAA exclusion was achieved with a continuous running 3-0 Prolene suture closure, without resection. Electrical insulation of the LAA was not assessed. All patients underwent ablation of the posterior left atrial wall with a Medtronic monopolar irrigated RF catheter (Medtronic, Minneapolis, MN). This device has an irrigated tip that allows the delivery of high energy (up to 20–30 W) without excessive perilesional tissue heating. The transesophageal probe was removed during ablation to avoid esophageal lesions by RF erogation. Considering the increased risk of circumflex artery damage and, in our experience, the limited advantage in terms of procedural success, the lesion lines towards the MV were not included in the ablation scheme.

In the postoperative period, atrial arrhythmias (AAs) such as AF, atrial tachycardia, or atrial flutter were treated with an amiodarone bolus of 150 mg, followed by the infusion of 1 mg/kg/h for 6 hours and then 0.5 mg/kg/h. Subsequently, in those patients who needed further treatment, amiodarone was prescribed with oral administration, starting with 600 mg daily for the first week, 400 mg daily for the second week, and then 200 mg daily. If amiodarone was contraindicated (thyroid, liver, lung, or ocular disease), patients were treated with sotalol 80 to 120 mg twice a day.

If pharmacological SR restoration was unsuccessful or if there was recurrence of AAs, electric cardioversion was attempted once prior to discharge. All patients were discharged on warfarin according to their CHA_2_DS_2_-VASc score and/or type of prosthetic valve. At the first follow-up visit, the anticoagulation regimen was modified in accordance with the type of mitral valve procedure and patient characteristics.

All patients underwent outpatient follow-up visits at 1 and 2 years from discharge, which included anamnestic, electrocardiographic, and clinical evaluations.

The efficacy endpoint of the study was rate of freedom from AF at discharge and at 2-year follow-up across the study population. At follow-up, cardiac rhythm was verified using electrocardiograms (ECGs) and 24-hour Holter electrocardiograms in case of suspicious symptomatology.

The added value of LAA exclusion as part of the rhythm control strategy in patients with AF history who have undergone MV surgery was also separately evaluated in subgroups according to preoperative AF duration (≤ or >1 year) and left atrial dimensions (ML-LAD≤ or >6 cm).

Atrial arrhythmia recurrence was defined as any episode of AF or atrial tachycardia (AT) lasting more than 30 seconds. A period of 12 weeks was assumed as a blanking period during which any atrial tachyarrhythmia episode was not considered as recurrence.

The safety endpoints were need of a permanent pacemaker (PMK), occurrence of stroke, necessity of secondary surgical exploration, and left circumflex artery or esophageal lesions.

### 2.1. Statistical Analysis

Continuous variables were expressed as mean ± standard deviation. Normally distributed continuous variables were compared using Student's *t*-test, and nonnormally distributed continuous variables were compared with Mann–Whitney test. Categorical variables were presented as numbers and percentages and were compared by Fisher's exact test or *χ*^2^ analysis. Comparison of arrhythmia recurrence between patients who did and those who did not undergo LAA surgical exclusion and of the results according to preoperative AF duration (≤ or >1 year) and ML-LAD (≤ or >6 cm) was performed using log-rank test, and Kaplan–Meier curves were generated. All statistical analyses were performed using SPSS statistical software (Release 26.0; SPSS Inc., Chicago, IL). A *p* value ≤ 0.05 was considered statistically significant.

## 3. Results

Population characteristics are shown in Table 1. Mean age was 65 ± 12 years (females: 64% and males: 36%). The mean ejection fraction was 55.9 ± 11%. All patients had documented AF with onset ranging from 1 up to 120 months before the index procedure and with a mean duration of 30.8 ± 1.6 months. Overall, 42% of patients had AF history ≤1 year, and 58% had AF history >1 year. At the time of the procedure, 40% of patients were in SR, whereas 60% presented with AF. The average ML-LAD, assessed during the preoperative transthoracic echocardiogram, was 5.2 ± 0.92 cm. The study population was divided according to ML-LAD into two groups: patients with ML-LAD ≤ 6 cm (*n* = 75) and patients with ML-LAD > 6 cm (*n* = 25).

Among all patients, 10% underwent MV replacement with a mechanical prosthesis and 29% with a biological prosthesis. MV repair was performed in 61% of the cases. The encircling of the PVs and LA posterior wall isolation were performed in all patients with the use of radiofrequency ablation. The LAA was excluded in 52% of the patients.

Extracorporeal circulation mean time was 90 ± 23 minutes, while mean aortic cross-clamp time was 71 ± 14 minutes. AFSA procedure mean time was 12 minutes.

Mean postoperative intensive care unit (ICU) stay was 48 hours, and mean hospitalization time was 12 days (ranging from 7 to 36 days).

Eighty patients (80%) presented AF during the postoperative period and were treated as described above. Overall, 85% of patients had been preoperatively treated with oral anticoagulants.

At discharge, 69% of patients were free from AF, with 62 patients in SR and 7 with paced rhythm. No statistically significant association was found among LAA exclusion and SR at discharge (Table 2). Indeed, 71.1% of 52 patients who underwent LAA exclusion and 66.6% of 48 patients who did not undergo LAA exclusion were in SR at discharge (*p*=0.628). Regarding the antiarrhythmic therapy, no differences were found at discharge among patients who underwent LAA exclusion (44 out of 52, 84.6%) and those who did not (40 out of 48, 83.3%) with *p* value = 0.538.

### 3.1. Efficacy Endpoint at 2-Year Follow-Up

**Figure 2:**
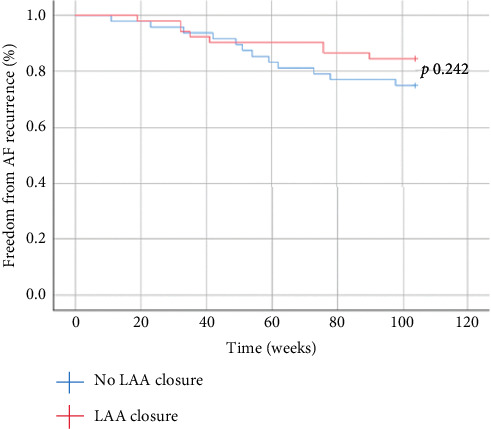
Freedom from AF recurrence in LAA and no-LAA patients at 2-year follow-up. At 2-year follow-up, there was no statistically significant difference in SR maintenance among patients who received and did not receive LAA surgical exclusion, 44 patients (84.6%) and 36 patients (75%), respectively (log-rank *p* 0.242). LAA: left atrial appendage; SR: sinus rhythm.

At 2-year follow-up, the overall freedom from AF was 80%, and there was no statistically significant difference in SR maintenance between patients who did and did not receive surgical LAA exclusion, with 44 out of 52 (84.6%) patients and 36 out of 48 (75%) patients in SR, respectively (log-rank *p*=0.242) (Figure 2).

At 2-year follow-up, the number of patients treated with AADs among patients who underwent LAA exclusion was 24 out of 52 (46.1%) and 18 out of 48 (37.5%) in patients who did not undergo LAA exclusion, with a nonsignificant difference in the use of AADs among groups (*p* value = 0.251).

### 3.2. Impact of Baseline Left Atrial Diameter on the Maintenance of the Sinus Rhythm

**Figure 3:**
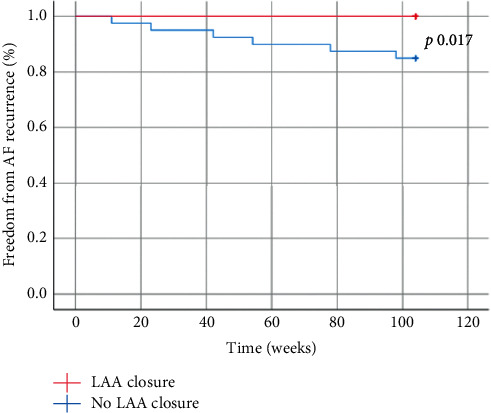
Freedom from AF recurrence in patients with ML-LAD < 6 cm at 2-year follow-up. At 2-year follow-up, there was a statistically significant difference in SR maintenance among patients with ML-LAD < 6 cm who received (red line) and did not receive (blue line) LAA surgical exclusion, 35 patients (100%) and 34 patients (85%), respectively (log-rank *p* 0.017). LAA: left atrial appendage; ML-LAD: medial-lateral left atrium dimensions; SR: sinus rhythm.

Since atrial enlargement is a well-known strong predictor of postprocedural AF recurrence [16, 17], we divided the population based on atrial size in order to investigate the potential benefit of LAA exclusion on AF recurrence in patients with and without advanced atrial remodelling. At discharge, 59 out of 75 patients (78.6%) with ML-LAD ≤6 cm were in SR compared to 10 out of 25 patients (40%) with ML-LAD > 6 cm (Table 2). A statistically significant difference was found among the two groups (*p* value ≤ 0.001). Overall, at 2-year follow-up, 69 out of 75 (92%) patients with ML-LAD >6 cm were in SR compared to 11 out of 25 (44%) patients with ML-LAD > 6 cm (*p* value ≤ 0.001). In the subgroup of 75 patients with ML-LAD ≤ 6 cm, 35 (46.7%) patients underwent LAA exclusion, while 40 (53.3%) did not. No differences in baseline characteristics were found (Table 3). No differences in rates of AF freedom were found at discharge with 29 out of 35 patients who underwent LAA exclusion in SR and 30 out of 40 patients who did not receive LAA exclusion in SR (*p* value = 0.407). A significant association between the maintenance of SR and the LAA exclusion procedure was noted at 2-year follow-up with rates of freedom from AF of 100% in patients with ML-LAD ≤ 6 cm and 85% in those with ML-LAD > 6 cm, respectively (log-rank test *p*=0.017) (Figure 3). The same analysis was performed on the subgroup of patients with ML-LAD > 6 cm. No significant association was found between the LAA exclusion procedure and AF freedom at 2-year follow-up. The efficacy endpoints at discharge and 2-year follow-up are summarized in Table 2.

### 3.3. Impact of Atrial Fibrillation Duration on the Maintenance of the Sinus Rhythm

At discharge, 29 out of 42 patients (69%) with AF duration ≤ 1 year were in SR, whilst 38 out of 58 patients (65.5%) with AF lasting > 1 year were also in SR. No association was found among AF duration at the time of the index procedure and sinus rhythm maintenance at discharge (*p* value = 0.711).

AF onset ≤ 1 year prior did not influence SR maintenance at 2-year follow-up. Similar rates of AF freedom between patients with AF history shorter than 1 year and those with longer history have been recorded at 2 years (85.7% vs. 75.8%; *p* value = 0.224).

### 3.4. Safety Endpoints

Seven patients needed permanent PMK implantation during hospitalization because of sinoatrial node (SAN) or atrioventricular node (AVN) dysfunction; none of the patients with valid rhythm at discharge showed SAN or AVN dysfunction at 1- and 2-year follow-up.

Postoperative stroke occurred in one patient. Two patients underwent surgical re-exploration due to acute bleeding, but neither of those were caused by the ablation. Neither left circumflex artery nor esophageal lesions were recorded.

## 4. Discussion

The main findings of the study are as follows:

1. AFSA provides good results in terms of SR maintenance during follow-up in patients with AF.
2. The beneficial effect of adding LAA exclusion at MVS is only seen in patients with ML-LAD ≤ 6 cm, pointing out the importance of timing in the treatment of AF. Early intervention to reduce the progression of AF-induced adverse atrial remodelling is crucial in achieving durable freedom from AF. Indeed, the detrimental effects of AF on atrial tissue progressively lead to atrial enlargement, fibrosis, and electrical and anatomical remodelling, lowering the success rate of rhythm control strategies. Our results underscore the need for early, aggressive rhythm control management of AF in order to prevent atrial remodelling before it becomes irreversible.

In our study, 80% of patients at 2-year follow-up were in SR, similar to the data reported in the literature [18–22]. The increase of 11% in terms of SR maintenance at 2-year follow-up compared to discharge demonstrates that rhythm control can be obtained even after several months from AFSA and is supported by results from other studies [23]. This trend seems not to be related to the effect of AADs as, in our cohort, there was a progressive reduction of AAD assumption over time. This increase of procedural success could be explained by good results of combined MVS + AFSA, which reduced LA myopathy [24, 25], while the reported success rate of MVS alone in restoring SR is very low (<10%) [26–28].

In our experience, a “minimal” approach (LA posterior wall ablation combined with PV isolation and MVS), compared to biatrial ablation and addition of an ablation line towards the MV, endorses Haissaguerre's theory according to which the most frequent AF trigger would be localized around the PVs [4, 29–31]. However, the LAA seems to trigger AF in 29% of patients, and it is the only site of recurrence in 8.7% of paroxysmal or persistent AF treated by catheter ablation [14]. In agreement, promising data have been published on the use of ablative non-PV strategies that target the LAA [32–36], notably ligation of the LAA [14, 37]. A systematic review, published in 2017, demonstrated that LAA isolation, in addition to standard targets of ablation, allowed the achievement of freedom from atrial arrhythmias without an increased incidence of complications [38].

Although there is no LA diameter cutoff to exclude patients for AFSA, many studies demonstrated higher rates of AF recurrence related to a severely dilated LA [20]. In a systematic review [18], twenty studies examined LA diameter as a predictor of AF recurrence, but very few patients had ML-LAD > 6 cm. Our subgroup analysis confirmed that ML-LAD > 6 cm is associated with AF persistence after AFSA. These poor outcomes are likely related to the atrial remodelling degree: the more advanced the atrial structural derangement, the less the probability of reverse remodelling and SR maintenance. At discharge, patients with diameter ≤ 6 cm had very high success rates compared to those with diameter > 6 cm: 69/75 (92%) vs. 11/25 (44%), *p* value ≤ 0.001. This suggests an early timing for intervention on MV defects with AF before the LA dilates excessively.

In agreement with [39], we achieved better results in patients with AF history < 1 year, compared to those with a longer one, although without statistical significance. However, ablation procedures also seem to improve SR maintenance in patients with long-standing persistent AF [40].

We excluded the LAA in 52 patients out of 100, according to the operator's preference, closing the LAA base with an internal continuous running suture, without resection or clear electrical insulation, and we looked back to check if excluding or not the LAA influenced the results in terms of SR maintenance.

Including all 100 patients, we did not find any difference at follow-up related to LAA surgical exclusion, and therefore, we investigated whether the exclusion might have changed the results in the four subgroups, divided according to AF onset (≤ or >1 year) and ML-LAD (≤ or >6 cm). The only subgroup which had statistically different results was the ≤6 cm ML-LAD group where the exclusion of the LAA allowed to achieve SR in 100% of patients at 2-year follow-up (*p*=0.017), while there was no difference at discharge (*p*=0.407). Such results may suggest the exclusion of the LAA in this subgroup of patients, even though some authors are worried about the pressure overload caused by volume reduction, especially when the LAA is particularly enlarged.

Concerning safety, ASFA combined with surgery seems to not increase the risk of stroke [39, 41]. In fact, we only observed 1 postoperative stroke case, probably due to thromboembolism after anticoagulant therapy interruption before PMK implantation.

Risk of PMK implantation is not entirely clear [22], but seems to be low. Our data suggest that only 7% of patients needed PMK implantation after AFSA + MVS. Furthermore, neither left circumflex artery nor esophageal lesions occurred, and we only had two cases of secondary surgical exploration because of postoperative bleeding.

### 4.1. Limitations

This is a retrospective, nonrandomized study in which the limited number of patients and the protracted enrolment period do not allow us to draw definitive conclusions about LAA exclusion effectiveness on AF recurrence. Although there were no differences in the subgroups' baseline characteristics, LAA exclusion was only performed in accordance with operator's preferences. In addition, the LAA isolation was anatomical: physically excluding it, without the corresponding established electrical isolation or imaging assessment. The choice of a ML-LAD of 6 cm as the cutoff value was arbitrary, although supported by similar values in the literature [14]. However, we cannot exclude that the cutoff value may be slightly different, and further studies will be required in order to identify the atrial enlargement threshold where LAA exclusion is beneficial. Moreover, only the midterm outcome was investigated. Longer follow-up will provide more definitive answers.

## 5. Conclusions

Our experience demonstrated that MVS + AFSA (limited to PV and LA posterior wall isolation) achieves excellent results in terms of SR maintenance, without increasing the risk of complications.

Overall, LAA exclusion did not influence results, but significantly increased the AFSA success rate at 2-year follow-up if the ML-LAD was ≤6 cm, while preoperative AF duration did not affect the maintenance of SR during follow-up. Such results may influence the selection of patients who are eligible for AFSA and may suggest an early timing indication for surgery (ML-LAD ≤ 6 cm), with associated concomitant LAA exclusion. It will be of primary importance to focus further attention on different aspects in the literature: (1) extensive literature review and prospective studies to identify the main predictors of the efficacy of atrial fibrillation ablation; (2) possible alternative sites that may improve the outcome of this technique, such as ablation of Bachmann's bundle [42]; (3) a comparison of the energies and strategies used.

We retrospectively analyzed 100 patients who underwent MVS + AFSA in our institution from January 2008 to June 2017. We compared the effectiveness of AFSA in patients who underwent LAA exclusion to those who did not. Moreover, we analyzed the role of preoperative AF duration (≤ or >1 year) and medial-lateral left atrial dimensions (ML-LAD) (≤ or >6 cm). The efficacy endpoint was freedom from AF at 2-year follow-up. The safety endpoints were need of a permanent pacemaker (PMK), secondary surgical exploration, occurrence of stroke, and left circumflex artery or esophageal lesions.

## 6. Clinical Perspective

The present study reports the clinical outcomes of AFSA.

With 30 years of follow-up, a low risk of progression to permanent atrial fibrillation was observed, with older age at diagnosis or the presence of subtle ECG abnormalities predictive of progression to permanent atrial fibrillation.

Overall survival and survival free from cardiovascular death or heart failure were not significantly affected by the presence of lone atrial fibrillation.

Stroke or transient ischaemic episode occurred only after the development of risk factors, including advanced age or hypertension.

The present long-term follow-up study provides evidence for the heterogeneous nature of atrial fibrillation and the important modulatory effects of comorbidities on its progression and complications. Because of the low risk of progression to permanent arrhythmia in young patients with isolated atrial fibrillation, the role of invasive therapies in lone atrial fibrillation needs to be studied carefully in randomized controlled trials.

In addition, screening for comorbidities is essential for this group because of the increased risk of complications on their emergence.

## Figures and Tables

**Table 1:** Baseline characteristics of the total study population and of the subgroups of patients who underwent and did not undergo LAA surgical exclusion.

Variables	Total population (*n* = 100)	Patients with LAA surgical exclusion (*n* = 52)	Patients without LAA surgical exclusion (*n* = 48)	*p* value
Age (mean, years)	65 ± 12	63 ± 10.5	66 ± 13	0.271
Females (*n*)	64	34	30	0.764
AF subtype				
Paroxysmal	31	17	14	0.674
Persistent	69	35	34	0.782
Type II diabetes (*n*)	11	7	4	0.413
Type I diabetes (insulin-dependent) (*n*)	9	5	4	1
Hyperlipidemia (*n*)	24	14	10	0.476
COPD (*n*)	9	5	4	1
Hypertension (*n*)	59	31	28	0.896
Smoking (*n*)	44	24	20	0.652
Previous CVA/TIA (*n*)	14	8	6	0.678
Chronic kidney disease (*n*)	33	18	15	0.721
NYHA III-IV (*n*)	46	21	25	0.168
History of endocarditis (*n*)	11	7	4	0.322
Warfarin preprocedural therapy (*n*)	85	44	41	0.911
Left ventricle ejection fraction (mean)	55.9 ± 11	54.6 ± 11.5	56 ± 9.2	0.341
Mitral valve disease				
Mitral stenosis	19	9	10	0.99
Mitral regurgitation	81	43	38	0.786
Mitral valve replacement with mechanical prosthesis (*n*)	10	6	4	0.743
Mitral valve replacement with biological prosthesis (*n*)	29	18	11	0.198
Mitral valve repair (*n*)	61	29	32	0.264

COPD: chronic obstructive pulmonary disease; CVA: cerebrovascular accident; NYHA: New York Heart Association; TIA: transient ischaemic attack. A *p* value < 0.05 was considered statistically significant.

**Table 2:** Efficacy endpoints at discharge and 2-year follow-up.

		Total number	LAA surgical exclusion	No LAA surgical exclusion	*p* value
Total population	At discharge (*n*; %)	69	37/52 (71.1)	32/48 (66.6)	0.628
	At 2-year follow-up (*n*; %)	80	44/52 (84.6)	36/48 (75)	0.242

Patients with ML-LAD ≤ 6 cm	At discharge (*n*; %)	59	29/35 (82.8)	30/40 (75)	0.407
	At 2-year follow-up (*n*; %)	69	35/35 (100)	34/40 (85)	0.017

Patients with ML-LAD > 6 cm	At discharge (*n*; %)	10	7/17 (42.2)	3/8 (37.5)	0.790
	At 2-year follow-up (*n*; %)	11	8/17 (47)	3/8 (37.5)	1

LAA: left atrial appendage; ML-LAD: medial lateral left atrium diameter; SR: sinus rhythm. A *p* value < 0.05 was considered statistically significant.

**Table 3:** Baseline features of patients divided according to the left atrial dimension and type of treatment. Statistical comparisons among groups are shown.

Variables	ML-LAD ≤ 6 cm		*p* value	ML-LAD > 6 cm		*p* value
	LAA surgical exclusion (*n* = 35)	No LAA surgical exclusion (*n* = 40)		LAA surgical exclusion (*n* = 17)	No LAA surgical exclusion (*n* = 8)	
Age (mean, years)	65 ± 4.22	65.3 ± 5.9	0.745	63 ± 7.4	65.7 ± 3.2	0.144
Females (*n*)	22	26	1	10	6	0.729
Type II diabetes (*n*)	3	5	0.713	2	1	0.543
Type II diabetes (insulin-dependent) (*n*)	3	3	1	2	1	0.543
Hyperlipidemia (*n*)	10	10	0.797	3	1	0.752
COPD (*n*)	2	5	0.438	1	1	0.823
Hypertension (*n*)	24	21	0.237	10	4	1
Smoking (*n*)	14	16	1	9	5	1
Previous CVA/TIA (*n*)	18	14	0.168	3	1	0.791
Chronic kidney disease (*n*)	13	12	0.625	5	3	1
NYHA III-IV (*n*)	17	18	0.819	7	4	1
History of endocarditis (*n*)	4	4	1	2	1	0.543
Warfarin preprocedural therapy (*n*)	30	33	0.762	16	6	0.475
Left ventricle ejection fraction (mean)	57.1 ± 6.3	55 ± 4	0.086	53.9 ± 2.6	51 ± 6	0.381
Mitral valve replacement with mechanical prosthesis (*n*)	4	3	0.699	2	1	0.543
Mitral valve replacement with biological prosthesis (*n*)	8	6	0.554	5	2	0.810
Mitral valve repair (*n*)	20	25	0.646	12	4	0.578

COPD: chronic obstructive pulmonary disease; CVA: cerebrovascular accident; LAA: left atrial appendage; ML-LAD: medial-lateral left atrium dimensions; NYHA: New York Heart Association; TIA: transient ischaemic attack. A *p* value < 0.05 was considered statistically significant.

## Data Availability

The data used to support the findings of this study are available from the corresponding author upon request.
